# The effect of physical exercise on social appearance anxiety among college students: the serial mediating role of social media addiction and self-objectification

**DOI:** 10.3389/fpubh.2026.1866510

**Published:** 2026-07-08

**Authors:** Ningxiao Tang, Kangyuan Zhang, Le Yu

**Affiliations:** School of Physical Education, Hunan University of Science and Technology, Xiangtan, China

**Keywords:** physical exercise, social appearance anxiety, social media addiction, self-objectification, serial mediating effect

## Abstract

**Background:**

Social appearance anxiety (SAA) has become a prominent mental health issue among Chinese college students, and it is particularly exacerbated in the mobile Internet era, marked by the pervasive use of social media. As an active health-promoting behavior, physical exercise is widely recognized as being able to effectively alleviate individuals’ mental health problems, yet its underlying psychological mechanisms remain inadequately explored. Therefore, this study is based on social comparison theory, this study aims to investigate the relationship between physical exercise and SAA among Chinese college students, and to examine the serial mediating role of social media addiction and self-objectification in this association.

**Methods:**

A convenience sampling method was adopted to administer a questionnaire survey among college students from universities in central China, yielding 415 valid responses. The measurement instruments included the Physical Activity Rating Scale-3 (PARS-3), Social Appearance Anxiety Scale (SAA), Bergen Facebook Addiction Scale (BFAS), and Self-Objectification Beliefs and Behaviors Scale (SOBB). Data analyses were performed using SPSS 27.0 and PROCESS 4.2 macro, and the Bootstrap method was adopted to test the serial mediating effects with 5,000 resamples.

**Results:**

Correlation analyses revealed significant bivariate correlations between all variables. Serial mediation analysis indicated that the total effect of physical exercise on SAA was −0.498, and the direct effect was −0.313, accounting for 62.85%. All three indirect paths were significant: the independent mediating effect of social media addiction was −0.080, accounting for 16.06%; the independent mediating effect of self-objectification was −0.083, making up 16.67%; and the serial mediating effect of social media addiction and self-objectification was −0.022, accounting for 4.42% of the total effect.

**Conclusion:**

Physical exercise helps reduce college students’ SAA directly, and offers indirect benefits by suppressing social media addiction and alleviating self-objectification through independent and serial mediating pathways. These findings enrich the understanding of the psychological mechanisms through which physical exercise influences SAA, provide empirical evidence to advance the dynamic development of social comparison theory, and offer practical and targeted approaches to promote mental health in higher education contexts.

## 1 Introduction

With increasingly intensified social evaluation mechanisms, appearance anxiety has displayed prominent social characteristics. A World Health Organization survey shows that social pressure and peer evaluation are major triggers of psychological problems among adolescents, and SAA, with focuses on others’ evaluations, is a concrete manifestation of this mechanism in the domain of physical appearance (1). Research by the United Nations Children’s Fund further shows that over 50% of adolescents are dissatisfied with their appearance, which is largely influenced by peer comparison and the reinforced appearance evaluation system on media (2). The American Psychological Association also points out that frequent appearance comparison on social media exposes individuals to others’ judgments, thereby significantly intensified anxiety (3). This phenomenon is especially prominent among college students. University years are a sensitive period of body image and self-concept development, during which individuals face multiple pressures from academic competition, interpersonal adaptation, and future planning. These pressures render this group highly vulnerable to psychological problems (4). In the mobile Internet era. Social appearance has become an important form of social capital and personal identity. College students are particularly prone to excessive concern about and worry over their appearance, thereby leading to SAA (5). One study found that SAA is highly prevalent among young people, with over 70% of college students in coastal South India expressing dissatisfaction with their social appearance (6). Meanwhile, a questionnaire survey administered to Chinese college students conducted by China Youth Daily indicated that 59.03% of respondents suffered from appearance anxiety (7).

SAA refers to the anxiety triggered by individuals’ fear of receiving negative evaluations from others concerning their overall physical appearance. Long-term exposure to such anxiety is likely to cause a series of psychological problems including depression (8). Empirical studies show that SAA is frequently a key source of impaired social functioning and interpersonal distress among college students (9). Several other studies have indicated that SAA significantly impairs self-esteem, self-efficacy, and academic functioning in college students, and may trigger extreme behaviors such as eating disorders (10, 11). Hence, it is crucial to conduct a comprehensive investigation into SAA among college students and devise effective intervention strategies.

As a positive health-promoting behavior, physical exercise can enhance cardiopulmonary function, reduce obesity, and lower the incidence of diseases (12). It is also widely regarded by researchers as an effective intervention to improve body image, alleviate anxiety, ameliorate depression, and prevent cognitive decline (13–15). Physical exercise yields numerous physical and psychological benefits, but college students generally engage in insufficient physical activity owing to the academic-oriented higher education environment and the instant gratification afforded by the Internet. Data from the World Health Organization indicate that approximately 30% of adults worldwide fail to meet the recommended physical activity guidelines, and this figure continues to rise (16). Meanwhile, a recent assessment indicated that only 45.9% of college students in the United States fully met physical activity standards (17). It is worth noting that decreased time spent on physical exercise is often accompanied by increased screen time. This behavioral substitution may cause college students to focus more on their physical appearance, thus triggering SAA (18). Moreover, unlike general anxiety, SAA is particularly prominent in the context where social media is highly prevalent (19). According to social comparison theory, college students’ constant exposure to idealized body images may exacerbate appearance-related distress when they spend extensive time to screens rather than physical activities (20). Therefore, it is essential to place the analysis within the interactive environment of social media and body image when exploring the positive psychological effects of physical exercise on SAA. Accordingly, by investigating the impact of physical exercise on SAA among college students, this study incorporates social media addiction and self-objectification to explore the relationships among these four variables. It further attempts to uncover the unique positive role of physical exercise in alleviating SAA, so as to provide more targeted theoretical and practical guidance for mental health interventions among college students.

## 2 Literature review and hypotheses

### 2.1 The effect of physical exercise on SAA

Physical exercise (PE) refers to physical activity performed by individuals to promote physical and mental health or enhance athletic performance. Compared with broadly defined physical activity, it emphasizes goal orientation and structured arrangement (21). As a positive lifestyle, engagement in PE regulates neurochemicals to relieve negative emotions, thereby sustaining mental health (22). One study suggested that PE can alleviate stress, anxiety, and other emotional disturbances among college students by boosting positive psychological traits such as resilience (23), with a particularly pronounced mitigating effect on social anxiety (24). As a specific subtype of social anxiety, SAA is also directly or indirectly influenced by PE. Women who participate in regular exercise such as Pilates present lower SAA and better mental health, and PE is a protective factor against negative behaviors induced by appearance anxiety among female college students (25). For male students, PE not only directly lowers SAA but also exerts indirect effects by strengthening reinforcing athletic identity, improving self-efficacy, and fostering positive body perception (26). Based on the above analysis, the relationship between PE and SAA may not be a purely linear one; the impact of PE may be exerted on SAA by directly and indirectly shaping other psychological and behavioral characteristics of individuals.

Accordingly, this study puts forward Hypothesis H1: PE exerts a negative predictive effect on SAA among college students.

### 2.2 The mediating role of social media addiction

Social media addiction (SMA) is a type of compulsive behavior defined by uncontrollable use of social media (27). This addictive behavior negatively impacts individuals’ physical condition through factors such as sleep disturbances (28), while simultaneously exacerbating depression, anxiety, and psychological distress, and undermines overall mental health by reducing self-esteem and fostering negative self-perception (29). The relatively loose management structure of higher education renders such addictive behavior particularly prevalent among college students (30). The prevalence of SMA among college students is roughly 18.4%, rising to 22.8% in Asia (31). Moreover, a strong theoretical link exists between SMA and SAA. Social comparison theory asserts that individuals tend to evaluate their self-worth through social comparisons with others. Individuals are repeatedly exposed to upward social comparisons on social media platforms filled with highly curated and idealized images. Studies have verified that addicted individuals exhibit significantly higher levels of SAA due to longer and more intensive social media use (32). These two variables are significantly correlated with each other. Furthermore, SAA and SMA levels are higher among women, individuals with a low-income perception, and adolescents (33). Selfie-taking behavior has particularly notable impact on anxiety (34). Overall, SMA heightens individuals’ appearance-related focus and social comparison, thereby exacerbating their SAA.

Recent studies indicate that PE may serve as an effective strategy for alleviating SMA, and higher levels of physical activity are consistently linked with lower risks of SMA (35). Engagement in PE can directly reduce time spent on social media and also diminish addictive tendencies through a series of psychological processes (36). From the perspective of psychological compensation mechanisms, the pleasure, sense of achievement, and social interaction afforded by PE can replace for the emotional gratification provided by social media, thereby diminishing individuals’ psychological dependence on virtual social interactions (37, 38). Meanwhile, PE exerts a protective effect by modulating the relationship between negative emotions and addiction. Regular and moderate PE is of significant value in alleviating psychological dependence on virtual social networks (39). Furthermore, studies have indicated that physical exercise is an important method for intervening in related addictive behaviors, and a longer intervention duration may lead to better outcomes (40). In summary, this study puts forward that PE has a significant positive impact on SMA among college students, and that SMA acts as a bridge in the development and worsening of SAA.

Accordingly, this study puts forward Hypothesis H2: SMA plays a mediating role in the relationship between PE and SAA.

### 2.3 The mediating role of self-objectification

The concept of self-objectification (SOBB) derives from objectification theory, which refers to the tendency for individuals to view themselves from an observer’s perspective as objects and to habitually monitor their body appearance (41). This theory posits that in cultural contexts with frequent appearance-related evaluation, individuals gradually develop persistent monitoring of their physical appearance and habitually check whether their bodies meet mainstream social aesthetic standards (42). Meanwhile, individuals with high levels of SOBB also demonstrate lower executive function and slower response speed in academic and occupational tasks (43), and may have difficulty maintaining a stable self-identity due to impaired self-continuity and sense of authenticity (44). Existing research has established a strong link between SOBB and SAA. Objectification theory indicates that individuals are highly likely to experience shame and anxiety upon perceiving that they fail to meet such standards when they constantly monitor their bodies and internalize external aesthetic standards as a basis for self-evaluation (45). A meta-analysis has verified a direct, moderate positive correlation between SOBB, body image disturbance, and SAA across various populations (46). In addition, the sense of self-worth and body image concerns have been found to play a mediating role in the relationship between SOBB and social anxiety (47). Overall, SOBB represents a key psychological factor to the development and exacerbation of SAA.

Existing research suggests that PE holds great potential for alleviating SOBB among women. The enhanced self-confidence and self-compassion acquired from consistent exercise can effectively reduce SOBB tendencies (48). Specific forms of exercise such as resistance training among adolescents have also been demonstrated to reinforce physical self-efficacy and overall self-worth, effectively diminishing excessive focus on external appearance and promoting the development of a positive self-concept (49). Available evidence indicates that the beneficial effect of PE on SOBB mainly resides in its transformation of the way individuals experience their bodies: it shifts attention from visual appearance monitoring to functional bodily experience (50). Furthermore, relevant studies suggest that PE helps alleviate appearance-based SOBB through repetitive and meaningful physical engagement (51). In short, this study contends that PE can lower individuals’ SOBB levels, which in turn relieves them from constant appearance monitoring and thereby reduces SAA.

Accordingly, this study puts forward Hypothesis H3: SOBB plays a mediating role in the relationship between PE and SAA.

### 2.4 The serial mediating role of SMA and SOBB

Multiple cross-sectional studies and systematic reviews have established a significant positive correlation between SMA and SOBB (52). Adolescents, college students, and women are particularly susceptible to such effects, and individuals who frequently use visual social platforms such as Instagram, TikTok, and Xiaohongshu exhibit higher levels of SOBB (53). Individuals with higher levels of SMA exhibit more severe self-comparison and dissatisfaction due to greater exposure to idealized appearance standards (54). In particular, sharing behaviors on social media directly prompt users to view their bodies as objects of others’ evaluation (55). Meanwhile, studies have indicated that frequent social media use enhances self-monitoring, leading addicted users to observe their bodies from an external perspective more frequently (56). In addition, SMA is significantly linked with psychological problems including stress, anxiety, depression, and low self-esteem among college students, which indirectly contribute to elevated SOBB (57).

Social comparison theory posits that in the absence of objective standards, individuals evaluate and adjust their self-perceptions, abilities, and emotional states by comparing themselves with others. Comparative behaviors can be classified into upward and downward comparisons (58). Individuals with a strong tendency toward upward comparison tend to choose targets who are superior to themselves when making appearance-related comparisons. The resulting gap often cause comparative self-evaluation, including ego threat and negative emotions (59). In the era of rapid social media development, this psychological process has been greatly magnified by social media platforms, exacerbating body dissatisfaction and appearance anxiety (60). Based on social comparison theory, PE can be considered as a protective factor. Regular PE improves physical fitness and self-efficacy, thereby diminishing the tendency for negative comparisons in social situations (61). A study among college students demonstrated that PE can effectively improve individuals’ self-perception and self-esteem, and relieve SAA and related social anxiety (23). Furthermore, social comparison theory classifies comparisons into intentional and unintentional types. Unintentional comparison is an automatic, habitual and passive process induced by situational cues. SMA can be interpreted as a key factor directing social comparison toward automatic comparison (62). College students initially choose comparison targets intentionally when using social media, but repeated social comparison will be consolidated into an automatic cognitive pattern characterized by constant self-monitoring (63). Moreover, social comparison theory also asserts that comparison can occur between the actual self and the internalized ideal self (64). Under conditions of excessive use of social media, images of others are gradually internalized as appearance standards in the context of SOBB. Subsequently, negative emotions and self-denial arising from perceived discrepancies facilitate the emergence and exacerbation of SAA (39).

**Figure 1:**
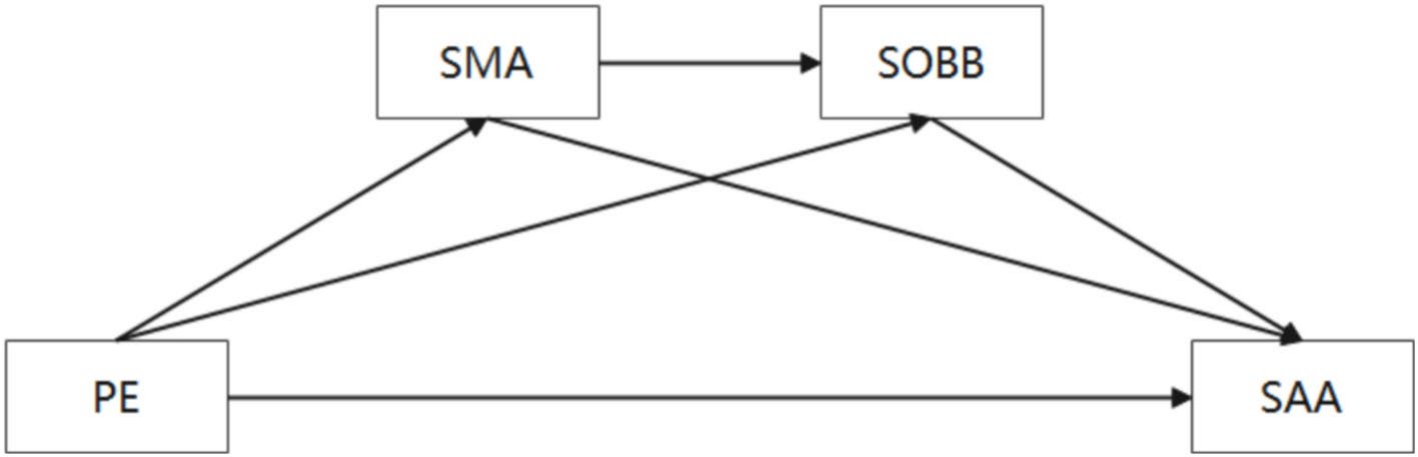
Hypothesized serial mediation model. PE, Physical exercise; SAA, Social appearance anxiety; SMA, Social media addiction; SOBB, Self-objectification.

Based on the above analysis, as well as previous discussions on the relationships among PE, SMA, SOBB, and SAA, this study puts forward Hypothesis H4: SMA and SOBB play a serial mediating role in the effect of PE on SAA. The serial mediation model established in this study is shown in Figure 1.

## 3 Methodology

### 3.1 Data sources

This study was carried out at universities in central China from January 2026 to March 2026. Central China is characterized by a large population and concentrated higher education resources. It is neither highly developed and internationalized as the eastern coastal regions nor as heavily supported by preferential policies as western China, thus representing a distinctive regional context within China. A convenience sampling method was adopted to recruit college students, with inclusion criteria comprising (1) Full-time undergraduate students; (2) No severe physical or mental disorders (e.g., disabilities, major depression), and exclusion criteria including (1) Inability to participate in PE due to special conditions or illnesses; (2) Having experienced major events in the past six months (e.g., bereavement); (3) Abnormal questionnaire responses (e.g., completion time < 1 min, inconsistent answers). Data were collected via Wenjuanxing, an online questionnaire platform analogous to Amazon Mechanical Turk. Respondents were informed in advance about anonymity, confidentiality, and the purpose of the survey. To validate instrument reliability, a pilot survey was conducted, yieling 100 valid responses. Cronbach’s alpha coefficients for all scales were greater than 0.8.

Before distributing the formal questionnaire, this study estimated the sample size using F tests in G*Power software (version 3.1.9.7). The parameters were set as follows: effect size $f^{2}$ = 0.15 (moderate effect), power (1 − β ) = 0.95, and significance level α = 0.05. The calculated minimum required sample size was 119 participants. A total of 440 questionnaires were collected in the formal survey. After screening based on the aforementioned inclusion and exclusion criteria, 415 valid questionnaires were retained, with an effective recovery rate of 94.3%, and exceeded the minimum sample size requirement. (Note: Participants who completed the questionnaire received a small incentive such as a voucher, which was unlikely to exert undue influence on responses.)

Table 1 presents the demographic characteristics of the sample. The sample comprised 206 male students (49.6%) and 209 female students (50.4%). With respect to household registration, 210 students (50.6%) were from urban areas and 205 (49.4%) from rural areas. In terms of grade level: 103 freshmen (24.8%), 90 sophomores (21.7%), 121 juniors (29.2%), and 101 seniors (24.3%). Regarding major: 166 humanities students (40.0%), 178 science students (42.9%), 57 sports and art students (13.7%), and 14 students in other fields (3.4%).

**Table 1:** Demographic characteristics of participants (*n* = 415).

Variable	Category	Frequency	Percentage
Gender	Male	206	49.6%
	Female	209	50.4%
Household registration	Urban area	210	50.6%
	Rural area	205	49.4%
Grade	Freshmen	91	21.9%
	Sophomores	95	22.9%
	Juniors	100	24.1%
	Seniors	98	23.6%
	Graduate and above	31	7.5%
Major	Humanities	166	40.0%
	Science	178	42.9%
	Arts and sports	57	13.7%
	Others	14	3.4%

### 3.2 Research tools

#### 3.2.1 PE questionnaire

The Physical Activity Rating Scale-3 (PARS-3) was developed and revised by Liang (1994) from Wuhan Sports University, China. This scale is well suited for Chinese college students and has been extensively adopted by researchers to measure the PE level of Chinese college students, yielding good validity (65). It evaluates individuals’ PE level from three dimensions: exercise intensity, exercise duration, and exercise frequency. The scale adopts a 5-point scoring system ranging from 1 to 5. PE level = Exercise intensity × (Exercise duration − 1) × Exercise frequency. Classification criteria are as follows: low exercise level: score ≤ 19; moderate exercise level: 20 < score ≤ 42; high exercise level: score > 42. The Cronbach’s alpha coefficient of this scale was 0.83 in a previous study among Chinese college students (66), whereas in the present study, it was 0.838.

#### 3.2.2 The Social Appearance Anxiety Scale

SMA among college students was measured using the Social Appearance Anxiety Scale (SAA) developed by Hart et al. (8). The scale comprises 16 items rated on a 5-point Likert scale from 1 (not anxious at all) to 5 (extremely anxious), with total scores ranging between 16 and 80. The SAA has demonstrated good reliability and validity in relevant studies and been Chinese practice (67). In the present study, the Cronbach’s alpha coefficient of this scale was 0.917.

#### 3.2.3 The 6-item Bergen SMA Scale

SMA among college students was assessed using the 6-item Bergen Social Media Addiction Scale (BSMAS) developed and revised by Andreassen et al. (68). The scale comprises 6 items rated on a 5-point Likert scale from 1 (strongly disagree) to 5 (strongly agree), with total scores ranging between 6 and 30. Higher scores reflect a greater severity of SMA. The BSMAS has been widely used among college student samples worldwide (69), and demonstrates good reliability and validity among Chinese college students (26). In the present study, the Cronbach’s alpha coefficient of this scale was 0.934.

#### 3.2.4 SOBB Beliefs and Behaviors Scale

SOBB among college students was assessed using the Self-Objectification Beliefs and Behaviors Scale (SOBBS) revised by Lindner and Tantleff-Dunn (70). Unlike the traditional Objectified Body Consciousness Scale, which developed primarily for female samples, this revised version is also validated for use to men (71). The scale comprises 14 items rated on a 5-point Likert scale from 1 (strongly disagree) to 5 (strongly agree), with the mean score serving as the indicator (range = 1–5). Higher scores indicate more frequent body surveillance and a greater level of SOBB. This scale has been widely adopted and validated among young people in China (50). The Cronbach’s alpha coefficient of this scale was 0.924 in this study.

### 3.3 Statistical method

Data analysis was conducted using IBM SPSS Statistics 27.0, with analytical procedures as follows: (1) Data cleaning: Cronbach’s alpha was calculated to evaluate the internal reliability of each subscale. After data collection, Harman’s single-factor test was conducted to evaluate potential common method bias. (2) Following the impot of clean data into SPSS, descriptive statistics were generated to summarize the demographic characteristics of the sample. Pearson correlation coefficients were calculated to explore the relationships among PE, SAA, SMA, and SOBB. (3) Regression analyses were performed for the four variables. Following data standardization, the serial mediation model was examined using Hayes’ PROCESS 4.2 macro. The significance level was set at p < 0.05.

## 4 Data analysis

### 4.1 Common method bias

Harman’s single-factor test was conducted to assess potential common method bias in the questionnaire survey. The results revealed four factors with eigenvalues greater than 1, and the first factor explained 35.9% of the total variance, below the 40% critical line. These findings suggest that there was no substantial common method bias in this study.

### 4.2 Correlation analysis of key variables

Pearson correlation analysis was conducted to explore the relationships among PE, SAA, SMA, and SOBB. As presented in Table 2, PE was negatively correlated with SAA ( r = −0.491, p < 0.01), SMA ( r = −0.375, p < 0.01), and SOBB ( r = −0.447, p < 0.01). SMA was positively correlated with SOBB ( r = 0.414, p < 0.01) and SAA ( r = 0.453, p < 0.01). SOBB was positively correlated with SAA ( r = 0.378, p < 0.01). The significant correlations among these variables provide a sound basis for the subsequent establishment and analysis of the serial mediation model.

**Table 2:** Descriptive statistics and correlation matrix of all variables (*n* = 415).

Variable	*M* ± SD	PE	SAA	SMA	SOBB
PE	25.11 ± 19.95	1			
SAA	46.69 ± 14.54	−0.491^**^	1		
SMA	18.88 ± 7.37	−0.375^**^	0.414^**^	1	
SOBB	2.96 ± 0.95	−0.447^**^	0.453^**^	0.378^**^	1

*p < 0.05, **p < 0.01.

### 4.3 Chain mediating effect test

As a result of the significant intercorrelations among PE, SMA, SOBB, and SAA, as well as the absence of multicollinearity, the data satisfy the statistical requirements for testing the direct and indirect effects of PE on SAA among college students. First, regression analysis was performed, and the results are presented in Table 3: In the SMA model, the standardized regression coefficient of PE predicting SMA was β = −0.380 ( p < 0.001), indicating that SMA decreased by 0.380 standard deviations with each 1-standard-deviation increase in physical exercise. PE was a significant negative predictor of SOBB ( β = −0.359, p < 0.001). In the SOBB model, PE and future orientation together explained 26% of the variance in SOBB ( $R^{2}$ = 0.260), whereas in the SAA model, the three variables jointly accounted for 34.9% of the variance ( $R^{2}$ = 0.349). SMA was a significant positive predictor of both SOBB ( β = 0.249, p < 0.001) and SAA ( p = 0.211, p < 0.001), and SOBB was a significant positive predictor of SAA ( β = 0.231, p < 0.001). After controlling for SMA and SOBB in the regression model, PE remained a significant negative predictor of SAA among college students ( β = −0.313, p < 0.001).

**Table 3:** Regression analyses of relationships among variables among college students.

Variable	SMA		SOBB		SAA	
	β	t	β	t	β	t
Gender	−0.182	−1.996^*^	0.005	0.058	0.025	0.309
Household registration	0.012	0.133	0.048	0.563	−0.007	−0.093
Grade	0.052	1.261	−0.076	−1.979^*^	−0.022	−0.610
Major	0.060	1.045	0.049	0.910	0.072	1.424
PE	−0.380	−8.321^***^	−0.359	−7.758^***^	−0.313	−6.728^***^
SMA			0.249	5.371^***^	0.211	4.681^***^
SOBB					0.231	4.961^***^
*R*	0.393		0.510		0.591	
$R^{2}$	0.155		0.260		0.349	
F	14.974		23.940		31.150	

*p < 0.05, ***p < 0.001.

After the data was standardized, the PROCESS 4.2 macro in SPSS was employed to control for demographic variables (gender, household registration, grade, and major), and a 95% confidence interval was adopted to test the chain mediating effect of SMA and SOBB between PE and SAA of college students. The PROCESS 4.2 macro is a custom plugin for the analysis of mediating and moderating effect, with settings as follows: Model 6 was specified, with PE = physical exercise, SMA = social media addiction, SOBB = self-objectification, SAA = social appearance anxiety, and Bootstrap sample size = 5,000. It is widely accepted in academia that a mediating effect is significant if the 95% Bootstrap CI [BootLLCI, BootULCI] does not include zero. As shown in Table 4, the total effect of PE on SAA was significantly negative ( β = −0.498, BootSE = 0.043, 95% Bootstrap CI [−0.583, −0.413]), indicating that greater PE levels were associated with lower SAA levels among college students. After adjusting for mediating variables, PE maintained a significant direct effect on SAA ( β = −0.313, BootSE = 0.047, 95% Bootstrap CI [−0.405, −0.222]), which accounted for 62.85% of the total. Accordingly, Hypothesis H1 was supported.

**Table 4:** Bootstrap test of mediating effects.

	β	BootSE	BootLLCL	BootULCI	%
Overall effect	−0.498	0.043	−0.583	−0.413	100%
Total mediating effects	−0.185	0.028	−0.247	−0.130	37.14%
PE → SMA → SAA	−0.080	0.020	−0.121	−0.044	16.06%
PE → SOBB → SAA	−0.083	0.020	−0.125	−0.046	16.67%
PE → SMA → SOBB → SAA	−0.022	0.006	−0.036	−0.011	4.42%

**Figure 2:**
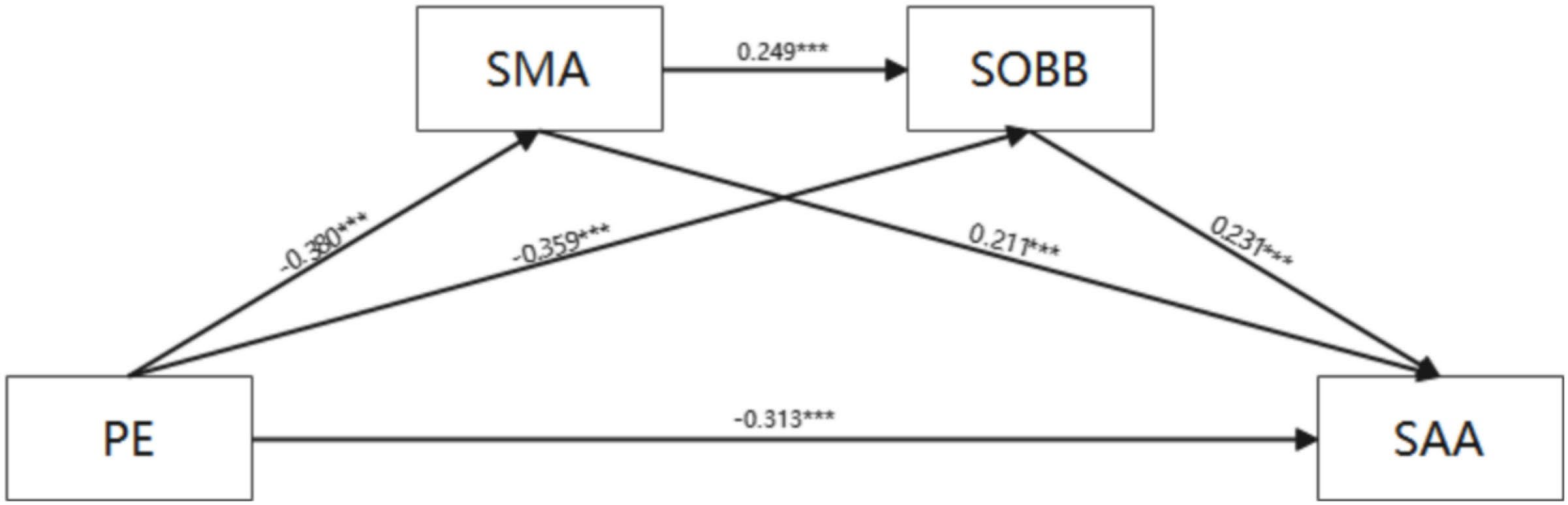
Chain mediating model of PE, SMA, SOBB and SAA among college students. PE, Physical exercise; SAA, Social appearance anxiety; SMA, Social media addiction; SOBB, Self-objectification. ***p < 0.001.

Furthermore, the results of the mediation effect test revealed that the Bootstrap 95% confidence intervals of the three indirect paths did not contain zero, indicating significant mediation. Specifically, the total mediating effect was −0.185 (95% Bootstrap CI [−0.247, −0.130]), accounting for 37.14% of the total effect. The first mediating path was PE → SMA → SAA, with an independent mediating effect of −0.080 (95% Bootstrap CI [−0.121, −0.044]), accounting for 16.06% of the total effect. Therefore, Hypothesis H2 was supported. The second mediating path was PE → SOBB → SAA, with an independent mediating effect of −0.083 (95% Bootstrap CI [−0.125, −0.046]), accounting for 16.67% of the total effect. Accordingly, Hypothesis H3 was supported. The third mediating path was PE → SMA → SOBB → SAA, with a chain mediating effect of −0.022 (95% Bootstrap CI [−0.036, −0.011]), accounting for 4.42% of the total effect. Accordingly, Hypothesis H4 was supported. These results demonstrate that PE can not only directly alleviate SAA among college students, but also indirectly reduce SAA via two independent paths (suppressing SMA and decreasing SOBB) and one chain path. The results from Table 4 were visualized as a chain mediation model, as illustrated in Figure 2.

## 5 Discussion

This study explored the mechanism underlying the link between PE and SAA among college students by investigating the mediating roles of SMA and SOBB. The results showed that physical exercise was both directly and indirectly related to college students’ social appearance anxiety, with the indirect relationship operating through the independent mediating effects of SMA and SOBB as well as their serial mediating effect. These findings partially supported Hypotheses H1–H4.

### 5.1 The effect of PE on SAA among college students

The results of this study revealed that PE was significantly negatively correlated with SAA among college students, and PE could directly and significantly reduce SAA ( β = −0.313, p < 0.001), indicating that each one-standard-deviation increase in PE was associated with a 0.313-standard-deviation decrease in SAA. This direct effect constituted 62.85% of the total effect, suggesting that PE serves as a strong direct protective factor against anxiety stemming from appearance concerns among college students, which is consistent with previous studies (72). Investigating the relationship among contemporary college students against the backdrop of wide social media penetration strengthens the practical significance of the findings, highlighting the unique value of PE in alleviating the psychological distress in the age of Internet. From a neurobiological perspective, regular PE facilitates the release of neurotransmitters such as endorphins, dopamine, and serotonin in the brain, thereby negatively predicting sensitivity to negative social evaluations (73). Also, PE helps modulate the function of the hypothalamic–pituitary–adrenal (HPA) axis and lowers baseline levels of stress hormones such as cortisol, providing more stable emotional basis (74). From a psychological perspective, the sense of accomplishment and self-efficacy derived from PE boost college students’ self-esteem directly, enabling them to develop stronger psychological resilience when confronted with appearance-related evaluations and reducing their vulnerability to anxiety elicited by external standards (75). Moreover, the positive body image fostered by PE helps avoid passively accepting judgments imposed by social aesthetic norms.

### 5.2 The mediating role of SMA

This study further revealed that SMA played a partial mediating role between PE and SAA among college students ( β = −0.080, p < 0.001). Specifically, PE not only showed a significant negative direct correlation with college students’ social appearance anxiety, but also exerted an indirect mediating effect on their anxiety by predicting lower frequency of and dependence on social media use, thereby supporting Hypothesis H2. Prior studies have confirmed that higher physical activity is significantly associated with lower risk of SMA, whereas SMA exacerbates SAA by enhancing individuals’ focus on their appearance. This study integrated these two established relationships and empirically tested the independent mediating of “PE → SMA → SAA” among Chinese college students for the first time. The effect size of this pathway (16.06%) and its significance underscore the value of virtual social behaviors in explaining the psychological benefits of PE. Social comparison theory posits that individuals evaluate self-worth by comparing themselves with others without objective criteria, and this psychological process is greatly amplified in the context of high social media prevalence (76). This study argues that SMA serves as a key transformer of social comparison from the intentional to the unintentional. For individuals with SMA, repeated and compulsive exposure to idealized body images leads them to habitually judge themselves against external aesthetic standards, thus generating persistent appearance dissatisfaction and anxiety (77). As indicated by prior findings, participation in PE can effectively predict this pathological process for college students. With reduced time spent on mindless scrolling, PE improves individuals’ subjective perception and acceptance of their own bodies, thereby negatively predicting SAA (72).

### 5.3 The mediating role of SOBB

The results demonstrated that PE negatively predicted SOBB levels among college students ( β = −0.359, p < 0.001), while SOBB positively predicted their level of SAA ( β = 0.231, p < 0.001). The results of the mediation analysis supported Hypothesis H3, indicating that SOBB played a significant mediating role in the relationship between PE and SAA ( β = −0.083, p < 0.001). The findings are consistent with prior research on the association between PE and SOBB. Mindful exercises such as yoga and Pilates, which emphasize internal bodily sensations and physical functionality, have been found to effectively enhance physical self-concept and reduce SOBB (78). Similarly, other studies have suggested that when individuals experience physical strength, flexibility, and a sense of bodily control during exercise, they tend to develop internal body self-identification rather than objectified perceptions rooted in external evaluations (48). The novel contribution of this study is that while physical exercise shows a significant negative correlation with SOBB-related body surveillance behaviors, it also exerts a mediating effect on SAA through SOBB, and this holds regardless of gender. Furthermore, the study extends previous findings by confirming that this association exists across a broader range of exercise modalities, and more precisely quantifies the variance explained by this indirect pathway in alleviating SAA among college students (16.67%). According to social comparison theory, engagement in PE reduces body self-monitoring associated with SOBB, thereby disrupting the pathway from SOBB to SAA. Prior studies have generally suggested that PE improves mental health by boosting self-esteem and self-efficacy (79). This further supports that the development of these positive psychological resources may be partly partially accounted for by reduced SOBB.

### 5.4 The chain mediating role of SMA and SOBB

When investigating the relationship between PE and SAA, this study identified a significant chain mediating effect through SMA and SOBB. The estimated chain mediation model revealed that SMA and SOBB jointly accounted for 37.14% of the total effect. Specifically, the independent mediated effect size of SMA was 16.06% ( β = −0.380 → β = −0.080), that of SOBB was slightly higher at 16.67% ( β = −0.359 → β = −0.083), and their chain interaction contributed an additional 4.42% ( β = −0.380 → β = −0.249 → β = 0.231). These findings indicate that PE not only negatively predicts SAA among Chinese college students through SMA, but also further accomplishes this process indirectly by negatively predicting SOBB. According to Social Comparison Theory, In the early stages of social media use, individuals may still consciously select comparison targets and conten, but as addiction appears, such comparison gradually becomes an automatized cognitive habit, which in turn promotes body self-monitoring (56). As noted, a core feature of SOBB is the habitual observation of one’s body from an external perspective. The significance of this pathway lies in the fact that PE can exert a sustained positive effect on SAA at two levels simultaneously: automated social comparison and habitual body surveillance. Furthermore, it is worth noting that although the serial mediating effect was significant, the regression coefficients were relatively small, suggesting that what we observed in this study may only represent one link among a broader range of underlying dynamic mechanisms.

Overall, by confirming the chain mediating effect of SMA and SOBB, this study This study has to some extent enriched the application contexts and psychological pathways of social comparison theory. Therefore, while designing PE intervention programs, beyond focusing on exercise volume, emphasis should also be placed on guiding college students in developing internal body identity through physical activity, thereby fundamentally reducing SAA.

### 5.5 Limitations

This study explored the internal mechanism underlying the effect of PE on SAA, thereby providing theoretical support for research focused on enhancing college students’ mental health. Although the data fit the model well, a few limitations should be noted: (1) This study employed a cross-sectional survey, which precludes rigorous inference of causal relationships among variables. Future studies should adopt longitudinal tracking or experimental designs; (2) The sample was recruited exclusively from central China. Future research should expand the sampling range to improve sample representativeness and external validity; (3) This study did not incorporate potential moderating variables that may impact the chain mediation pathway, such as personality traits, social support, and types of physical exercise. Future studies could construct moderated mediation models to further examine variations in the strength of the mediating effect under different conditions.

## 6 Conclusion

SMA and SOBB partially mediate the relationship between PE and SAA among college students. Additionally, SMA and SOBB exert a chain mediating role between PE and SAA. Specifically, PE exerts a direct effect on SMA and an indirect effect on college students’ SAA through the sequential chain pathway of SMA and SOBB.

## Data Availability

The raw data supporting the conclusions of this article will be made available by the authors, without undue reservation.
